# Cosmetic crimes in dromedary camels: a critical overview on these illegal manipulations and its hidden consequences

**DOI:** 10.3389/fvets.2025.1632022

**Published:** 2025-07-14

**Authors:** Mohamed Tharwat

**Affiliations:** Department of Clinical Sciences, College of Veterinary Medicine, Qassim University, Buraidah, Saudi Arabia

**Keywords:** animal welfare, camels, economics, ethics, tampering

## Abstract

This review highlights the emerging issue of illegal tampering in dromedary camels, particularly in the context of camel festivals where aesthetic alterations are performed to enhance appearance. These manipulations—often conducted by unqualified individuals—raise serious ethical and animal welfare concerns. Common techniques include mechanical and chemical modifications such as lip stretching, use of local anesthetics to alter facial expression, ear trimming, and injections of substances like silicone, fillers, and botulinum toxin (Botox) to modify head profiles. Hormonal treatments, including testosterone and growth hormone, are administered to induce exaggerated physical traits, often at the cost of fertility and health. Detection of tampering is increasingly challenging due to evolving methods and requires experienced personnel. Visual assessment remains the primary diagnostic step, followed by imaging techniques (e.g., ultrasonography, radiography, thermography) and hormonal assays. Economically, tampering distorts market values by misleading buyers on key animal traits, contributing to significant information asymmetry and undermining pastoral economies. It also introduces serious welfare issues, often carried out without veterinary oversight, resulting in pain, infection risk, and long-term functional impairments. Beyond health and economic implications, tampering undermines cultural integrity, erodes trust in livestock markets, and threatens food security in camel-dependent communities. Addressing this issue requires a multidisciplinary strategy involving scientific research, policy enforcement, community engagement, and education. Standardized inspection prostocols and mandatory certification can strengthen veterinary oversight and deter malpractice. In conclusion, the rise of illegal tampering in dromedary camels presents complex challenges to animal welfare, market integrity, and societal trust. A coordinated and ethically grounded response is essential to safeguard both animal and human livelihoods.

## Introduction

The Arabian camel (*Camelus dromedarius*) holds significant historical and cultural importance in the Middle East and Africa (1). Often regarded as a symbol of endurance and adaptability, it has been essential for survival in the harsh desert environment (2). The domestication of the camel and its longstanding relationship with the peoples of the Arabian Peninsula are central to understanding the region’s history and the evolution of camelid species (3). Camels became indispensable to the Bedouins, playing a crucial role in the development of ancient trade routes—especially the incense trade—which connected the Arabian Peninsula with the Mediterranean and beyond (4). Adapted to extreme heat and arid conditions, camels can travel long distances across barren deserts without water (5). Their ability to conserve water, withstand high temperatures, and carry heavy loads made them ideal for traversing desert landscapes (6). Additionally, their capacity to store fat in their humps allows them to survive extended periods without food, while their thick fur provides protection from intense sun and blowing sand (7). Their broad, cushioned hooves enable efficient movement across soft desert sand without sinking. These unique physiological features make camels exceptionally well-suited to desert life, where few other animals can survive (8).

Beyond their utilitarian roles, camels are deeply embedded in Arabian cultural heritage. They are prominent in poetry, religion, and social status (3). The famed camel caravans were not only essential for trade but also symbolized wealth and prestige (4). Reflecting this cultural significance, the Camel Club of Saudi Arabia established the King Abdulaziz Camel Festival (KACF). Held annually in December in the southern deserts of Al-Dahna near Riyadh, the festival aims to promote public awareness, support camel-related industries, and position camels as an economic asset for the Kingdom. It also highlights the developmental and economic benefits camels offer (9). At the conclusion of the KACF, a closing ceremony is attended by the King of Saudi Arabia or the Crown Prince, where winners receive prizes totaling up to 300 million Saudi riyals (approximately $80 million). Due to the high monetary stakes, some owners have resorted to illegal methods to enhance their camels’ appearances, such as lip injections, lip stretching, and nose modifications (10). These practices, which pose serious risks to the physical and psychological well-being of camels, are strictly prohibited by the KACF’s medical committees. Nevertheless, such cosmetic tampering is often performed before the festival by some owners attempting to beautify their animals (11).

To detect and control these illegal practices, the KACF organizing committee employs a medical team composed of specialized veterinarians throughout the event. Strict penalties are enforced for violators, including disqualification, bans from current and future events for both owners and their camels, and reporting to legal authorities for further action (9, 11–14). This review aims to shed light on the consequences of newly emerging illegal tampering practices in dromedary camels, with particular emphasis on their economic and animal welfare implications.

## The aesthetics of camel beauty contests: standards and commercial implications

**Figure 1:**
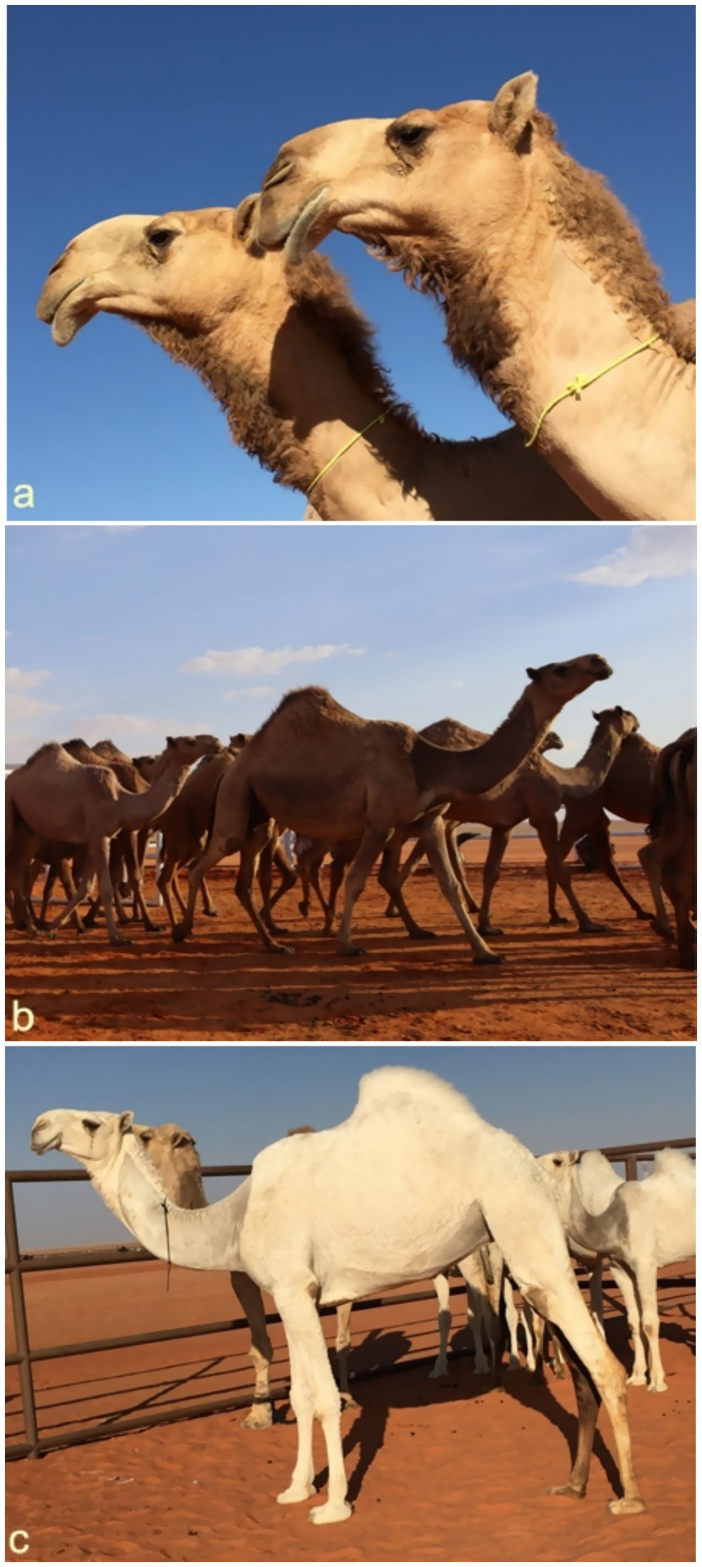
Some beauty standard in camels. The lips are long and cover the teeth, and the head is large. The eyelashes are long, and the nose is long and wide. The ears are also short and pricked backwards **(a)**. The neck is long and thin, and moving forward in height and the leg bones are large and long **(b)**. The hump lies to the back of the camel near the root of the tail **(c)** [*Reproduced from* (11)].

While traditionally associated with human participants, beauty contests have expanded to include animals—most notably camels (9). These competitions follow a codified set of aesthetic criteria used by specialists to evaluate camels’ physical attributes. Traits such as stature, height, and proportions are central to judgments of beauty, with emphasis placed on natural features over artificial enhancements. The increasing prevalence of cosmetic interventions in camels—primarily targeting the lips and ears—is largely driven by market dynamics. Owners seek to align their animals with idealized standards to increase commercial value (10). Camels exhibiting rare or highly desirable traits, such as distinctive eye color or specific coat patterns, can fetch prices in the millions (9, 12, 13). Beauty criteria differ slightly across camel breeds but generally prioritize a large head, long lips covering the teeth, wide and elongated nose, and long eyelashes (15). The ears should conform to breed-specific preferences: Majahatir camels typically have short, backward-facing ears, while Majaheem camels are expected to have longer, forward-oriented ones. A long, slender, upright neck and robust leg bones are also favored. Foot shape and size, tail length, and the positioning of the hump—ideally near the tail base—further distinguish prize-winning animals (11). These beauty standards reflect a structured aesthetic system and underscore the growing commercialization of camel breeding. As cosmetic procedures become more common, debates continue over authenticity, tradition, and economic influence in this culturally significant practice (14) (Figure 1).

To provide context and clarify the contribution of the current review, a comparative summary with previously published work is presented here. While the earlier article by Tharwat and Al-Hawas (11) offered a general overview of cosmetic tampering in dromedary camels—focusing mainly on beauty standards, tampering methods, and materials used—it did not address the broader implications of such practices. In contrast, the present review offers a comprehensive and multidisciplinary examination of illegal cosmetic manipulations, highlighting their animal welfare implications, economic and societal consequences, and ethical concerns. Notably, this review also introduces and illustrates the diagnostic utility of ultrasonography, radiography, and thermography in detecting various forms of tampering, with practical examples and annotated figures to support veterinary professionals and regulatory authorities in the field.

## Artificial enhancement and ethical concerns in camel beauty contests

**Figure 2:**
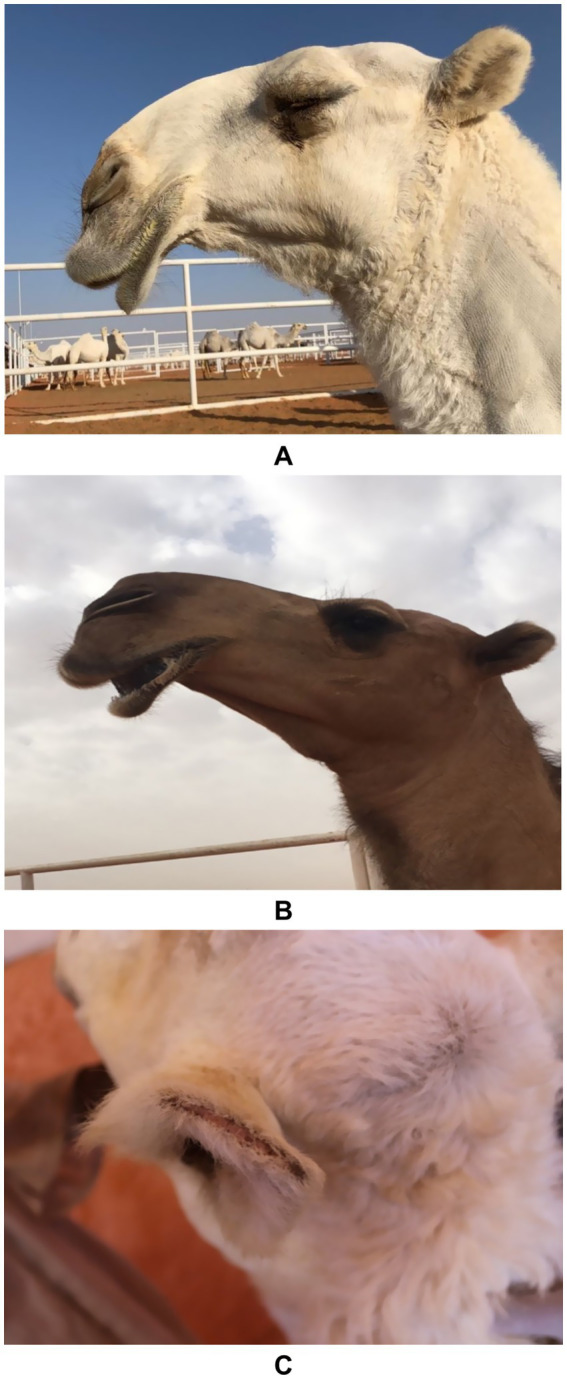
Artificial methods of tampering in dromedary camels. Image **(A)** shows enlargement, protrusion and stretched lips in a female camel due to stretching the lips and then pulling them outward. Image **(B)** shows drooping of upper and lower lips in a female camel due to continuous stretching and daily massage. Image **(C)** shows clipping or trimming the ears in a female camel in a trial to make the ears perkier [*Reproduced from* (11)].

Driven by lucrative prizes, some camel owners resort to artificial enhancements to align animals with prevailing beauty standards. These practices are frequently performed by unqualified individuals, posing ethical and welfare concerns (9). Common techniques include mechanical manipulation of the lips—such as binding with rubber bands, applying pressure with plastic to induce swelling, and daily stretching or massage—to achieve exaggerated drooping. Local anesthetics are also used to relax facial muscles, altering the camel’s natural expression (11). Invasive procedures have increased in popularity, including ear trimming, and the injection of substances like silicone, fillers, and Botox into the lips, nose, and jaw to create a more prominent and symmetrical head profile. Botox is particularly favored for enhancing the pout and overall facial size (16). Additional fraudulent practices include artificial pigmentation to alter coat color and the use of banned topical substances such as oils and sugars to enhance shine and texture (Figure 2). These interventions undermine competition fairness and highlight the need for stricter regulation and enforcement in camel beauty festivals (12). A summary of the common tampering types, methods, side effects, and diagnostic tools in dromedary camels are listed in Table 1.

**Table 1:** A summary of the common tampering types, methods, side effects, and diagnostic tools in dromedary camels (9, 10, 12, 14).

Type of tampering		Method	Potential side effects	Diagnostic tools
1	Lip and nose region filling	Injection of filler material to enlarge lips and the nose region	Pain, lip enlargement, infection, granulomatous reactions	Visual inspection, ultrasonography, thermography
2	Lip binding	Binding both lips with a rubbery belt and pulling them outward	Pain, lip swelling, necrosis, gangrene	Visual inspection, ultrasonography, thermography
3	Lip stretching	Stretching and daily massage of lips	Pain, inflammation, erosions, abrasions, wounds, flaccidity	Visual inspection, ultrasonography, thermography
4	Botox injection	Botox injections in the lips to decreases nerve indicatives and thus induce relaxation of the lips	Inflammation, tissue reactions, swelling, infection, necrosis, gangrene, bruising at the injection site	Visual inspection, ultrasonography thermography
5	Ear trimming	Surgical removal or shaping of ear cartilage o make them shorter	Inflammation, infection, pain, disfigurement	Visual inspection, comparison with breed standards
6	Local anesthesia	Topical or regional anesthetics, to encourage laxity in the lips	Transit or permeant lip paralysis, infection, inflammation	Visual inspection, needle nerve stimulation, low-voltage electrical stimulation
7	Nostril enlargement	Surgical incision to widen nostrils for aesthetic breathing flair	Nasal infections, respiratory issues	Endoscopy, imaging (ultrasound, X-ray/CT, thermography)
8	Muscle Enhancement (e.g., neck or hump augmentation)	Silicone or other injectable implants, oil injections	Granuloma, infection, inflammation, systemic toxicity	Ultrasound, palpation, foreign material detection
9	Hormonal manipulation	Injection of hormones such as testosterone or growth hormone to enhance muscle and bone growth	Organ dysfunction, abnormal muscle and bone growth, behavioral changes, reproductive abnormalities, protrusion of the clitoris and dulla	Blood tests, hormone level monitoring,
10	Dyeing and pigmentation	Use of ink or dyes to change coat color	Color fading	UV light, dye residue analysis
11	Surgical alteration of hump	Shaping through resection or implants	Pain, altered gait, infection, systemic inflammation	Ultrasound, X-ray/CT, visible asymmetry

**Figure 3:**
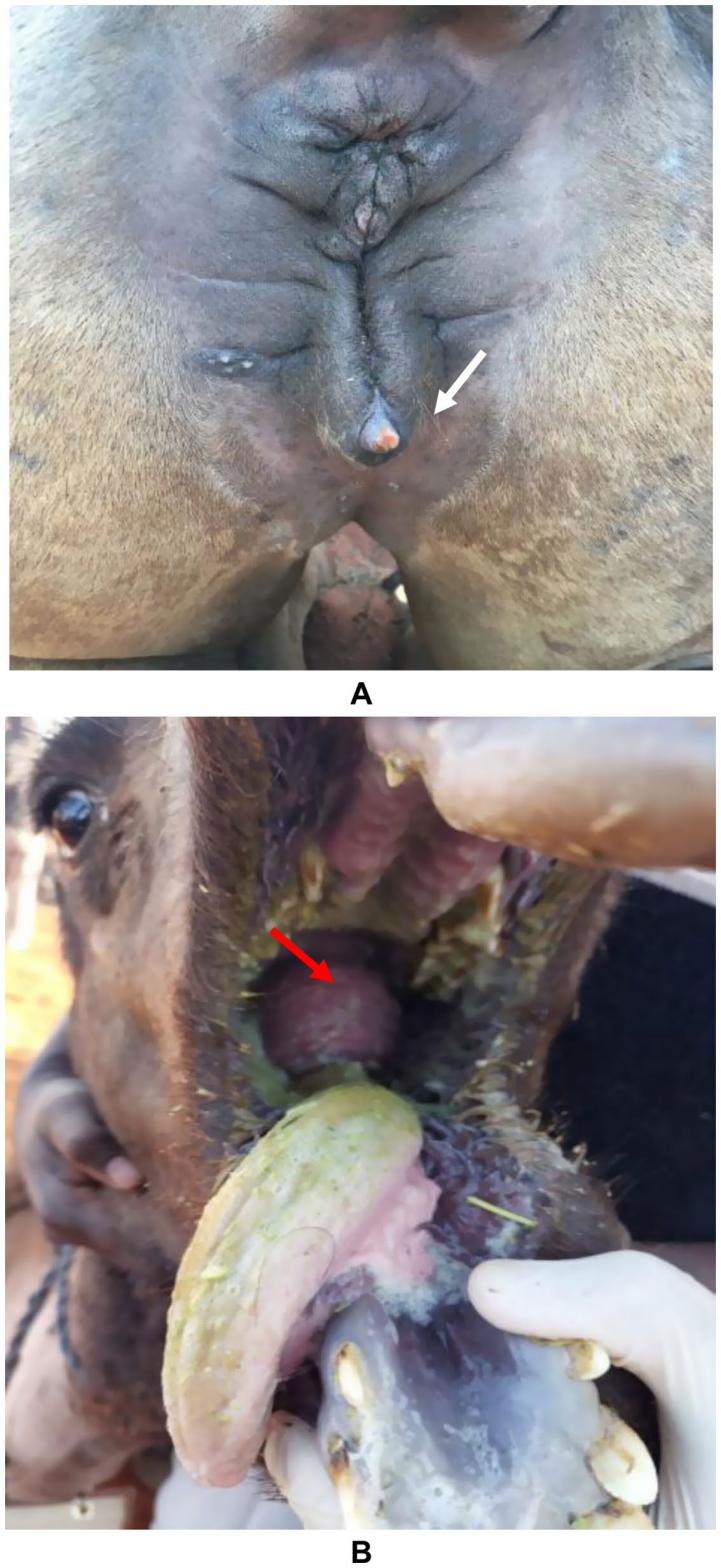
Side-effects of hormone injection in camels. Image **(A)** shows an enlarged clitoris (white arrow) in a female camel and image **(B)** shows the development of the dulla (red arrow) in a yearling female camel [*Reproduced from* (12)].

A minority of camel owners administer hormonal treatments to young camels to artificially modify their physique, aiming to accelerate growth, increase muscle mass, and enhance head and bone size. Commonly used hormones include testosterone and growth hormone (GH), with limited use of estrogen and progesterone. Hormones are used to treat certain health conditions in animals caused by endogenous hormonal deficiencies (13). However, their misuse can result in abnormal clinical signs. Administration of testosterone or GH leads to exaggerated somatic growth and altered bone development, though these acquired traits are not heritable. Hormonal injections cause both external and internal changes, including premature udder enlargement, clitoral hypertrophy, altered libido, social withdrawal, facial edema, voice changes, growth retardation in juveniles, and various dermatological lesions (11). In female camels, masculinization is common, particularly with early administration, manifesting as behavioral changes and pronounced male characteristics, including dulla enlargement and protrusion. Thus, these interventions generally produce observable changes such as enlarged head and neck, elongated bones, increased musculature, clitoral hypertrophy, and a higher incidence of infertility in females (17–19) (Figure 3).

## Incidence of cosmetic crimes of tampering among dromedary camels

**Figure 4:**
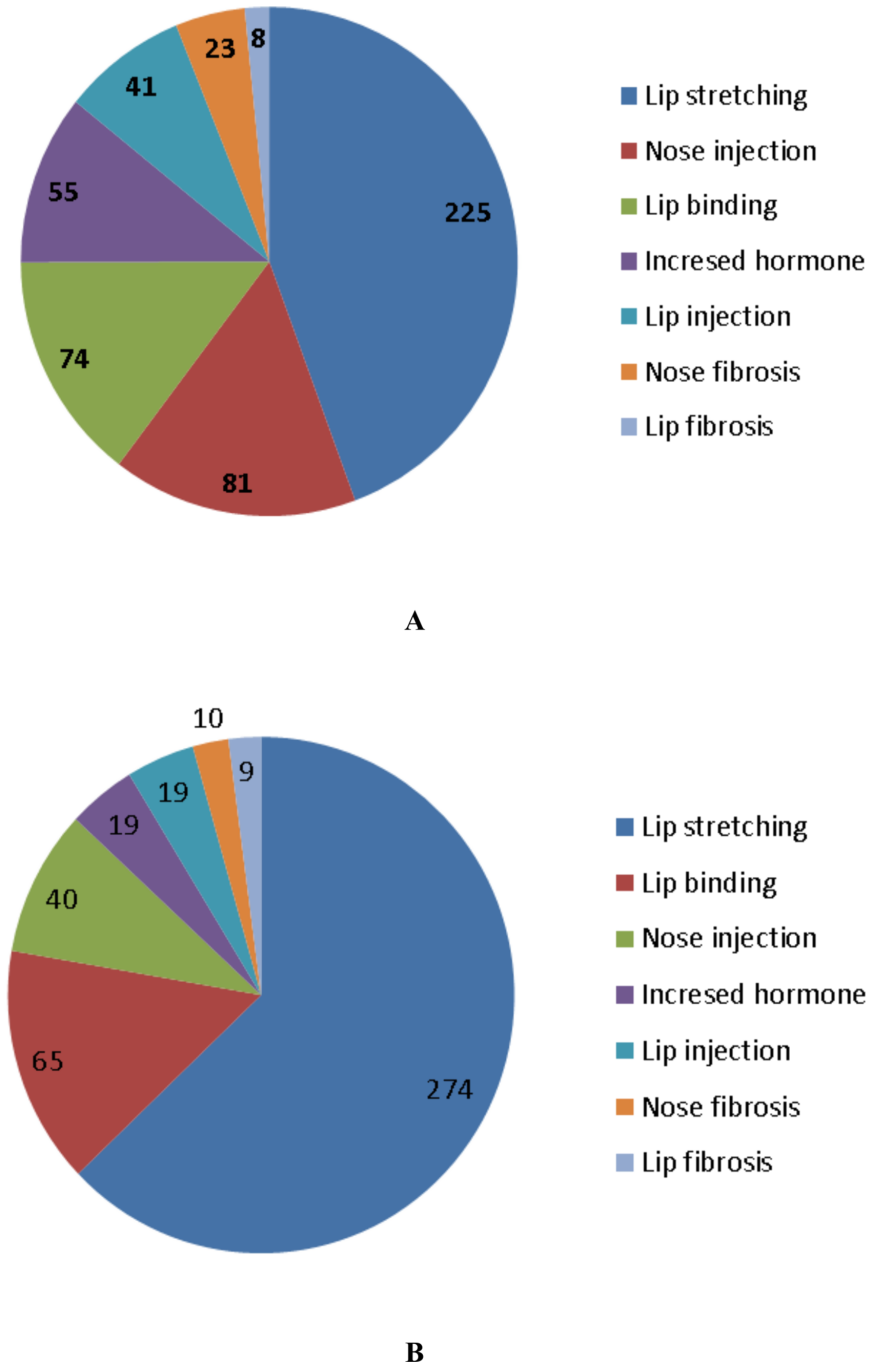
Different forms of tampering in dromedary camels. In image **(A)**, 1035 camels were examined. Different types of tampering were detected in 518 camels and included stretching of the lips, injection of the nose by a filler material, binding of the lips, increased testosterone level, injection of the lips by filler material, fibrosis of the nostril, and fibrosis of the lip. In image **(B)**, 11,350 camels were examined. The types of tampering detected in 433 camels included stretching of the lips, binding of the lips, injection of the nose by a filler material, increased testosterone level, injection of the lips by filler material, fibrosis of the nostril, and fibrosis of the lips [*Reproduced from* (12)].

A total of 12,385 dromedary camels (12,080 females and 305 males) were examined for evidence of illegal tampering procedures. The examination was conducted at two levels: external (physical inspection) and internal (using diagnostic tools). The investigation took place in Saudi Arabia during the 7th KACF, held in Riyadh from December 28th, 2022, to January 12th, 2023 (12). During initial external examination, 507 out of 1,035 camels (49%) exhibited signs of tampering. Of these, 500 were females and 7 were males. The most prevalent adverse manipulation was lip stretching, identified in 225 camels (43.4%), followed by nasal filler injections in 81 camels (15.6%), lip binding in 74 camels (14.3%), elevated testosterone levels in 55 camels (10.6%), lip filler injections in 41 camels (7.9%), nostril fibrosis in 23 camels (4.4%), and lip fibrosis in 8 camels (1.5%) (12). Examination of another group consisting of 11,350 camels, 436 camels (3.8%) were identified with evidence of tampering. Of these, 421 were females and 15 were males. Lip stretching was the most frequent alteration, found in 274 camels (63.3%), followed by lip binding in 65 camels (15%), nasal filler injections in 40 camels (9.2%), elevated testosterone levels and lip filler injections in 19 camels each (4.4%), nostril fibrosis in 10 camels (2.3%), and lip fibrosis in 9 camels (2.1%) (12) (Figure 4).

In retrospective study evaluating medical records of KACF during the third, fourth, fifth, sixth and seventh seasons and detecting different cosmetic methods among 53,090 dromedary camels, four primary tampering methods were identified. All the four practices showed a significant upward trend over the five seasons. Filler use increased from 0.126% in the 3rd season to 0.606% in the 7th (11). Similarly, lip stretching rose from 0.034 to 2.212%, lip binding from 0.025 to 0.525%, and hormone use from 0.008 to 0.129%. Overall, tampering prevalence increased markedly from 0.193% in the 3rd season to 3.496% in the 7th season. This surge in cosmetic tampering is likely driven by the substantial financial incentives awarded to winners, elevated social prestige among tribal communities, and the honor of participating in final ceremonies attended by high-ranking Saudi leadership (11). The findings of the later study highlight a significant rise in unethical cosmetic practices in dromedary camels over recent KACF seasons that pose health risks and may result in lasting complications.

## Methods for detecting tampering

Detecting tampering in camels requires experienced practitioners, as techniques continue to evolve, posing significant diagnostic challenges. Visual inspection remains the initial and most crucial step, typically followed by clinical evaluations such as ultrasonography, radiography, infrared thermography, or laboratory analyses of hormone levels. Ultrasonography is a non-invasive technique that can accurately detect and localize subcutaneous filler materials. The procedure has proven to be an essential diagnostic tool in dromedary camel medicine for evaluating thoracic and abdominal organs and diagnosing various intra-abdominal disorders (17–42, 57–61). Moreover, ultrasound has shown utility in the antemortem diagnosis of abdominal masses in camels (14, 37, 40, 43).

**Figure 5:**
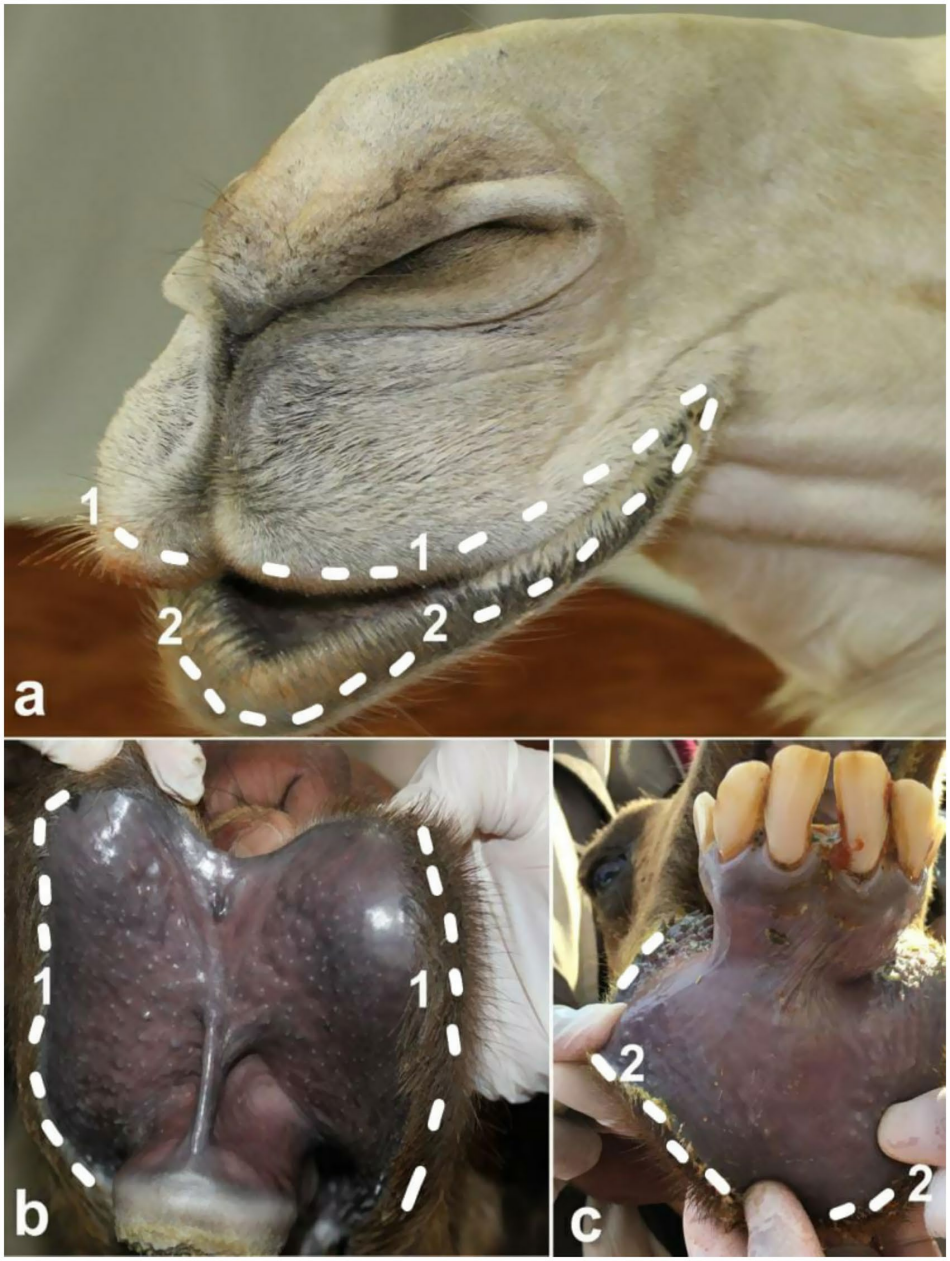
Mapping of the lips in a healthy camel pointing to different locations where injected fillers are commonly injected. The external surfaces of the injected upper and lower lips are shown in image **(a)** while the internal surfaces of the upper and lower lips are shown in images **(b,c)**, respectively. 1 represents the edges of the upper lips where thickness and filler injection were found (dashed line); 2 represent the edges of the lower lip where thickness and filler injection were detected (dashed line) [*Reproduced from* (10)].

**Figure 6:**
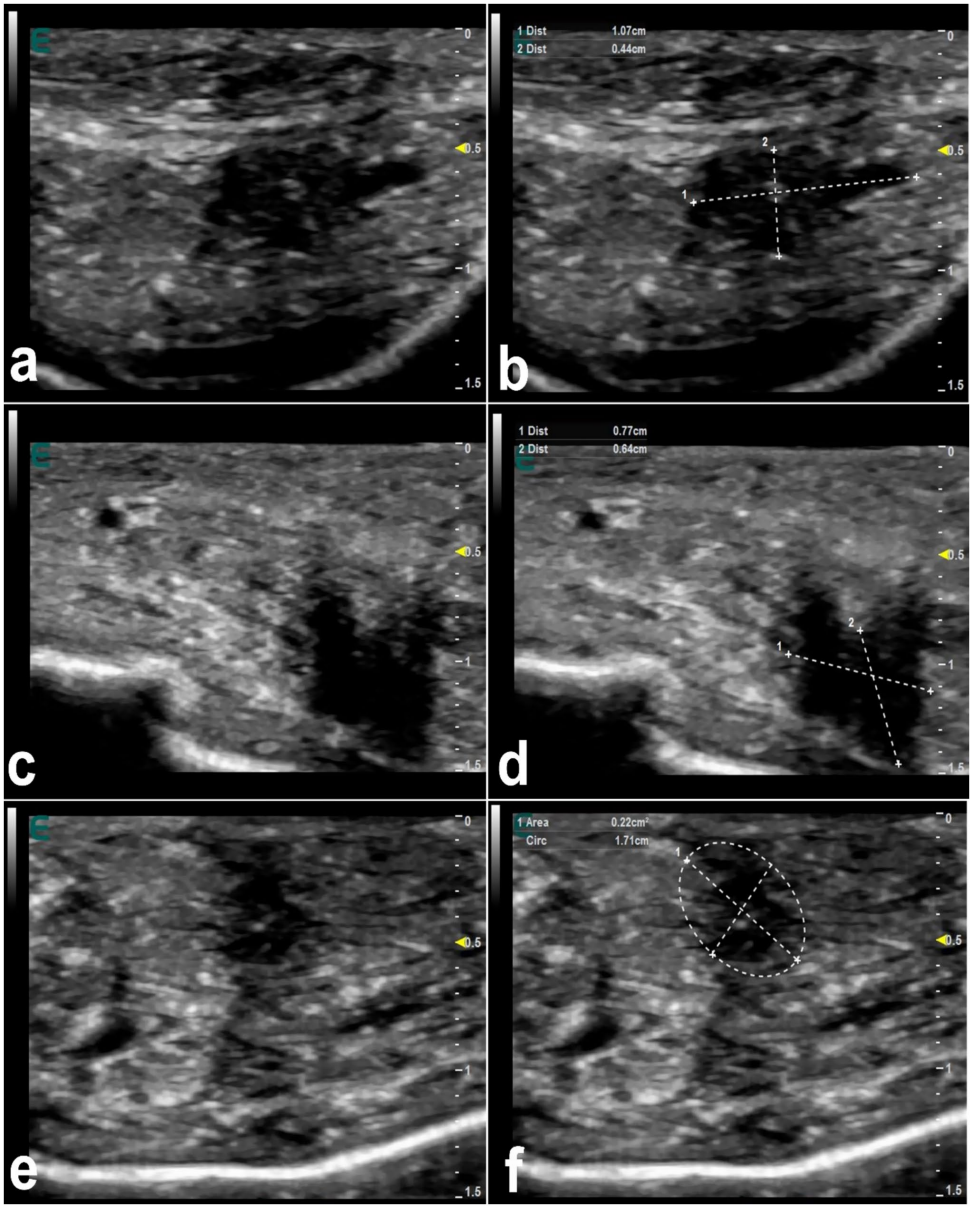
Ultrasonographic appearance of injected fillers in the upper lip of 3 female dromedary camels **(a,c,e)**. The injected filler appears anechoic to hypoechoic and was imaged in the form of small or large, single or multiple deposits. It is difficult to distinguish the subcutaneous tissue from the dermis. Images **(b,d,f)** represent a schematic representation of the images **(a,c,e)**, respectively [*Reproduced from* (44)].

**Figure 7:**
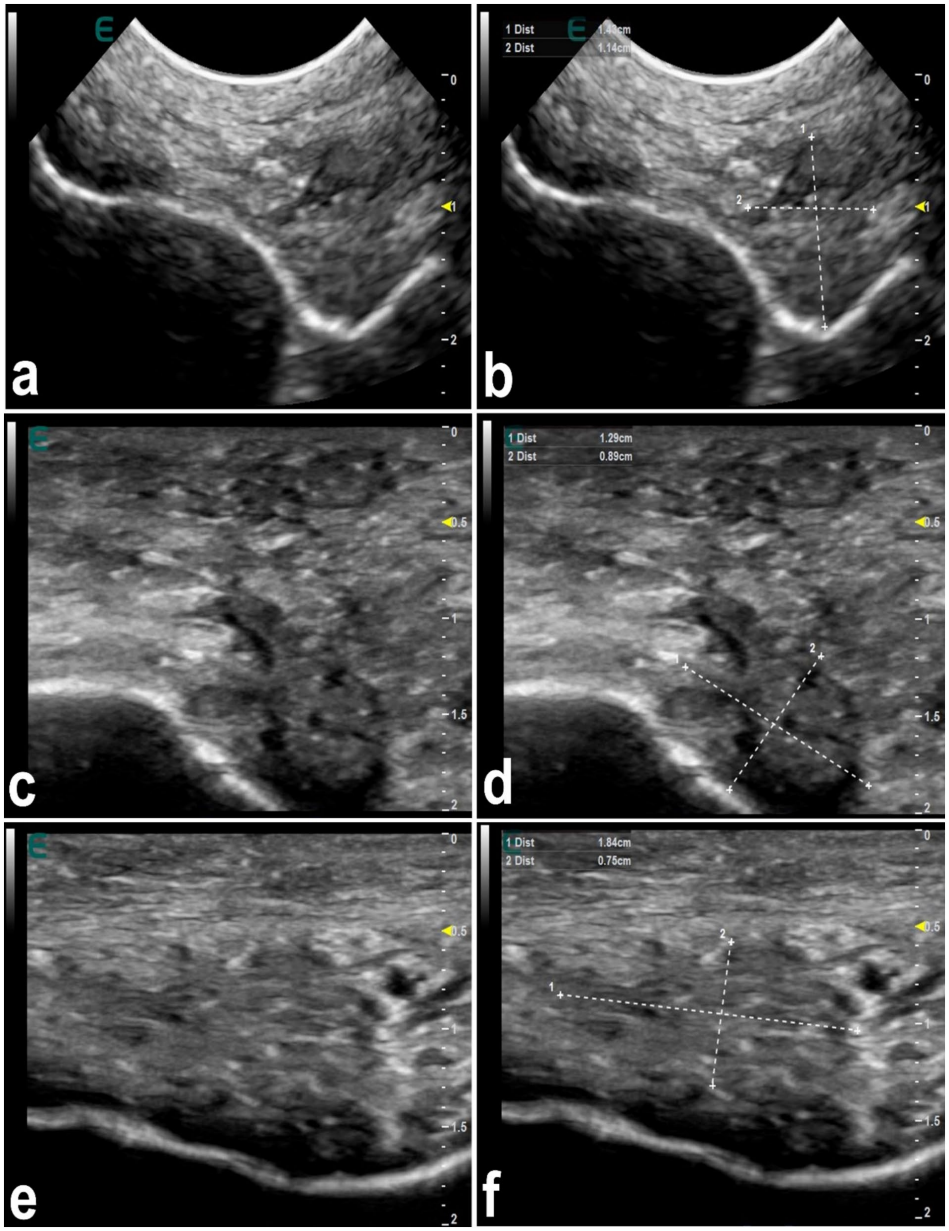
Ultrasonographic features of hardened tip of the lips in 3 dromedary camels with injected fillers **(a,c,e)**. The injected filler appears hypoechoic and the scanning patterns of the injected lips appear heterogeneous. Images **(b,d,f)** represent a schematic representation of the images **(a,c,e)**, respectively [*Reproduced from* (44)].

**Figure 8:**
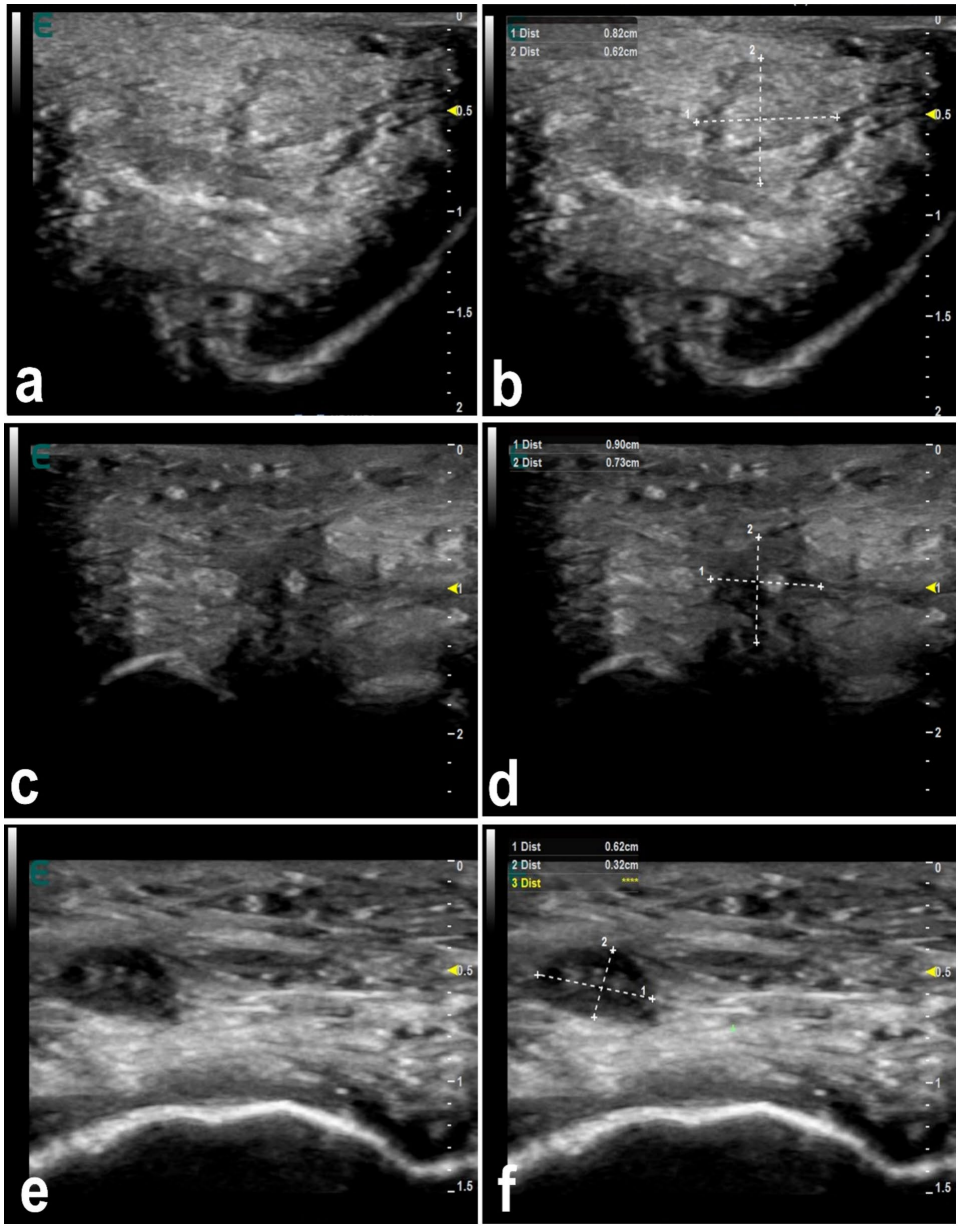
Ultrasonographic features of lip nodules in 3 camels as a result of filler injections **(a,c,e)**. They appear as either isoechoic with a hypoechoic rim or hypoechoic with an echogenic center. Images **(b,d,f)** represent a schematic representation of the images **(a,c,e)**, respectively [*Reproduced from* (44)].

Figure 5 shows mapping of the lips in a healthy camel showing different locations where injected filler materials are commonly injected. In healthy camels, the dermis appears hyperechoic, contrasting with the hypoechoic subcutaneous tissue (16). Filler substances, even those approved by regulatory agencies, can cause in humans detectable changes in tissue structure (63). Injected fillers in dromedary camels typically appear as anechoic or hypoechoic areas on ultrasound, presenting as single or multiple deposits of varying size. In affected camels, the boundary between the dermis and subcutaneous tissue becomes indistinct (Figure 6). Fillers in the firm edges of the lips exhibit hypoechoic signals, with the overall sonographic pattern of injected lips appearing heterogeneous (Figure 7). Lip nodules are visualized as either isoechoic structures with a hypoechoic rim or as hypoechoic lesions with an echogenic center (Figure 8).

**Figure 9:**
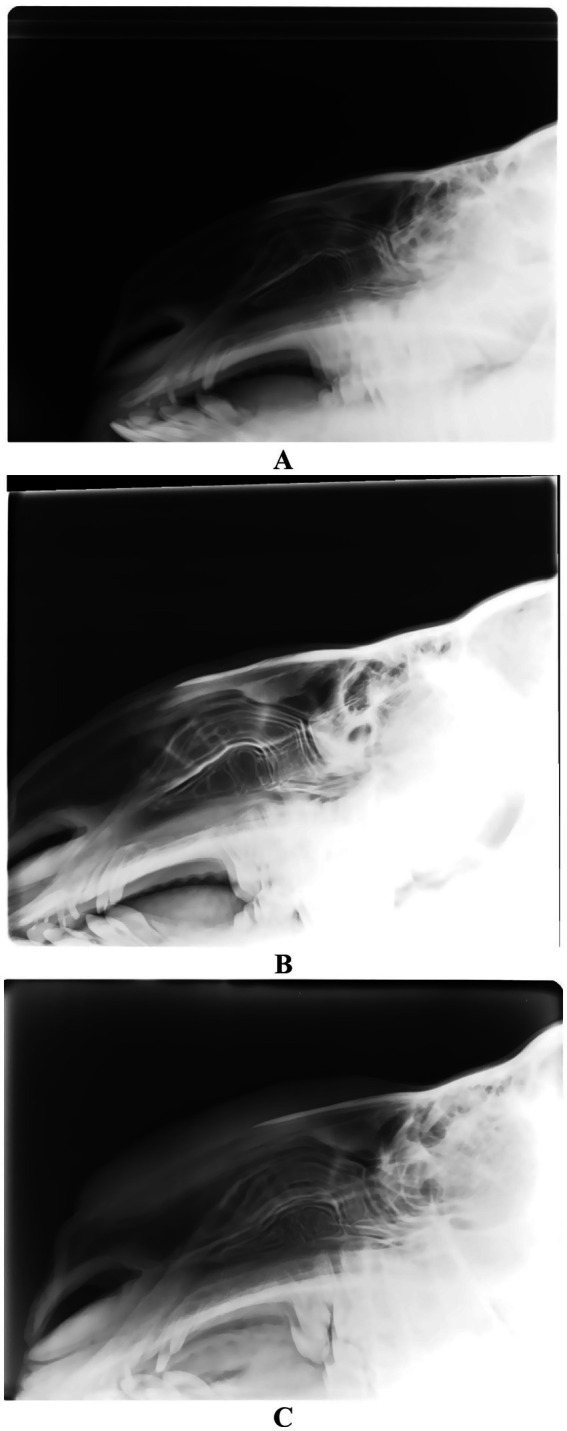
Lateral radiographic view of 3 camels injected with perinasal region fillers. Image **(A)** shows a healthy camel with no injections. Images **(B,C)** show camels with moderate and large quantities of filler injection, respectively. Please not the swelling of the surrounding soft tissues anterior and posterior to the nasal cartilage [*Reproduced from* (14)].

Radiographic evaluation of camels administered cosmetic fillers in the perinasal area demonstrated varying degrees of soft tissue enlargement. This was attributed to the injection of moderate to substantial volumes of filler material both anterior and posterior to the nasal bone (14). Notably, there were no observable alterations in the nasal bones or cartilage when compared to non-injected, clinically healthy camels. The filler material appeared as a soft-tissue density, presenting a gray tone on radiographic imaging within the perinasal region (14) (Figure 9).

**Figure 10:**
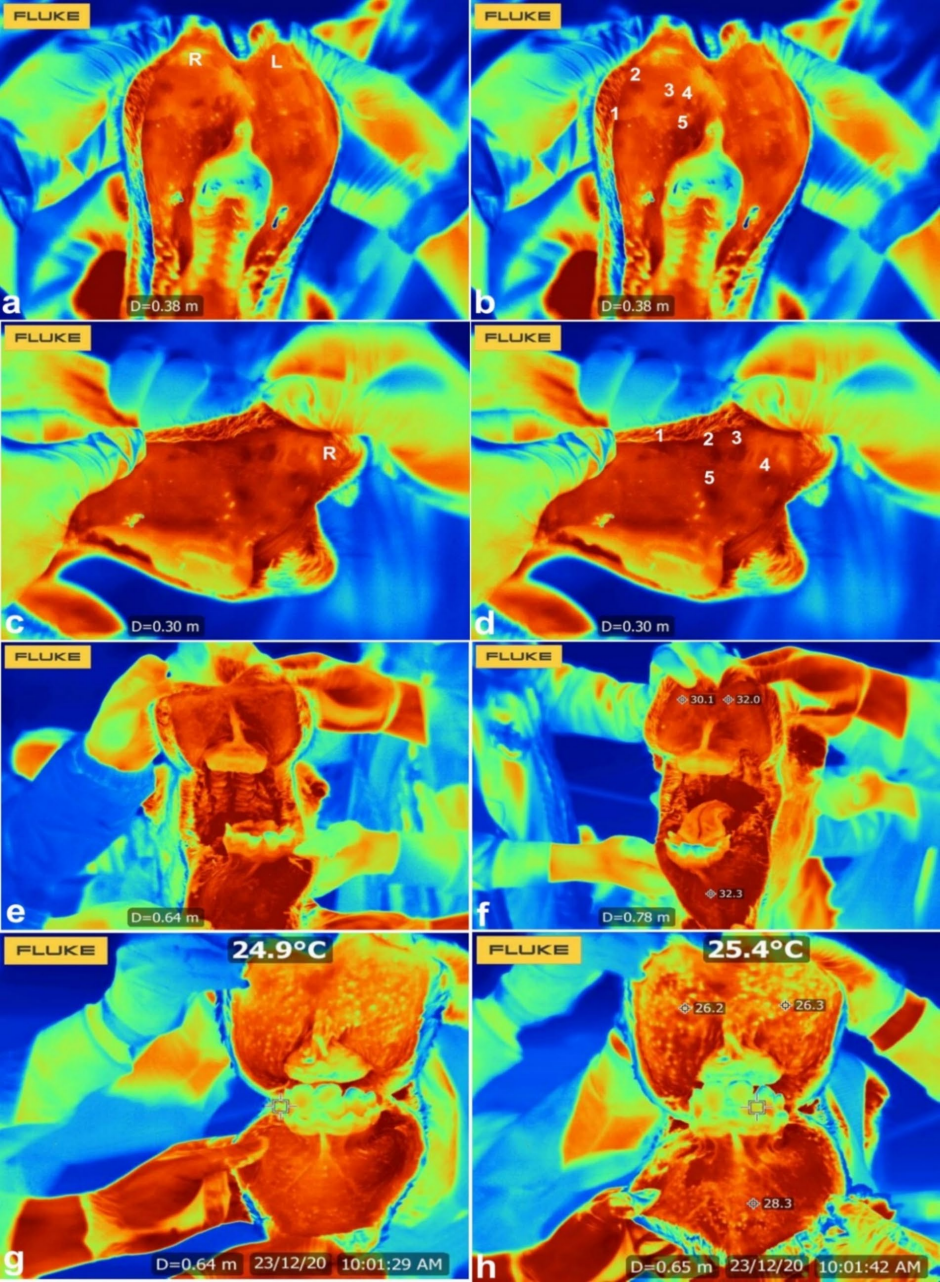
Infrared thermography of injected and stretched lips. Images **(a–d)** show injected upper lip in a camel where 5 injected sites (1–5) were detected and appeared darker than the surrounding tissue. Images **(e–h)** shows infrared thermography of stretched lips in a camel. The mucosal surface in the stretched lips appeared lighter and heterogeneous compared to the darker and homogenous pattern in the non-stretched lips. R = upper right lip; L = upper left lip [*Reproduced from* (44)].

Infrared thermography has revealed distinct thermal patterns associated with lip augmentation complications. Commonly observed features include localized swelling, firmness at the vermilion borders, and the formation of multiple dense nodules. Thermal imaging from the cutaneous surface demonstrates that the treated regions exhibit reduced thermal emission, appearing cooler than adjacent untreated areas. Similarly, on the mucosal side of the upper right lip, the injection sites also display lower temperature readings, contrasting with the warmer surrounding tissue. Notably, when the lips are stretched, mucosal thermographic images reveal a brighter and more heterogeneous thermal distribution, whereas non-stretched lips present a uniformly darker and more homogeneous pattern (44) (Figure 10).

## Economic aspects of tampering

In dromedary camels, tampering presents a complex economic issue involving a spectrum of direct and indirect costs and benefits that have substantial effects on pastoral and livestock-dependent economies. Tampering—defined as illicit interventions such as cosmetic enhancements or physical modifications intended to increase the commercial attractiveness of camels—creates distortions at both the production and market levels (9). These cosmetic crimes are typically designed to mislead buyers regarding critical attributes such as age, health status, or productivity, thereby artificially inflating prices and contributing to information asymmetry within the market. Although such practices may offer short-term financial gain to sellers, they can lead to longer-term negative outcomes, including loss of consumer confidence, deterioration of market credibility, and greater regulatory oversight (13, 14). Moreover, tampering may compromise animal health and performance, ultimately resulting in decreased productivity and economic returns due to reproductive inefficiencies and disease complications (10). On a broader scale, widespread tampering threatens the integrity and sustainability of the camel trade, particularly in regions where camels are integral to food security and rural livelihoods. Consequently, addressing the economic impact of tampering is essential for informing sound policy measures, fostering fair trade standards, and enhancing the long-term viability of camel husbandry systems (12, 16).

## Welfare effects of tampering and legal frameworks

In humans, cosmetic medicine encompasses a broad range of non-surgical and minimally invasive procedures aimed at enhancing physical appearance, improving self-esteem, and addressing age-related changes (45–47). Popular treatments include Botox injections, dermal fillers, chemical peels, and laser therapies, which target concerns such as wrinkles, volume loss, skin pigmentation, and scarring (48). Unlike reconstructive procedures—which are performed to correct congenital defects or trauma-related deformities—cosmetic treatments are typically elective and motivated by personal aesthetic goals (49). The field has expanded rapidly in recent years, driven by technological advancements, growing societal acceptance, and increasing demand for minimally invasive beauty solutions (50). Despite these trends, ethical concerns remain, particularly regarding the influence of social media, body image pressures, and the accessibility of procedures that may have significant psychological and physical consequences (49). In contrast, cosmetic medicine in veterinary practice remains relatively limited. Among dogs, two of the most commonly performed cosmetic procedures are tail docking and ear cropping. Although these practices have a long history, they continue to generate ethical and medical debate over their necessity and justification (51). In horses, cosmetic interventions are typically limited to the treatment of traumatic injuries or inflammation, particularly involving the eyes (52, 53).

Tampering in camels raises critical welfare issues that span physical, physiological, and behavioral aspects. Such practices—often involving invasive or deceptive alterations—are frequently performed without veterinary oversight and in unsanitary conditions, resulting in significant pain, risk of infection, and long-term health consequences. These modifications impair the animals’ capacity to carry out basic biological functions such as eating, moving, and reproducing, thereby compromising their overall health and welfare (13, 14). Beyond the immediate physical damage, tampering can trigger chronic stress responses, evidenced by increased cortisol levels, behavioral changes, and weakened immune defenses. The continued application of these harmful interventions reflects a broader neglect for animal ethics and constitutes a violation of recognized welfare standards (Tharwat and Al-Sobayil, 2021). Moreover, the psychological strain and diminished quality of life endured by affected camels are of particular concern in regions where camels play a crucial role in both cultural heritage and economic livelihoods. Mitigating the welfare impacts of tampering necessitates an integrative strategy that combines public education, stringent veterinary oversight, and effective enforcement of animal welfare legislation to promote humane treatment and ensure sustainable camel husbandry (12, 16).

Legal frameworks addressing animal welfare are central to curbing cosmetic tampering in dromedary camels. In Saudi Arabia, the Animal Welfare Act (54) prohibits acts that inflict unnecessary pain, suffering, or harm to animals, explicitly aligning with international standards set by the World Organization for Animal Health (WOAH) (55). Despite such legislation, enforcement gaps and limited veterinary oversight during major camel events often hinder effective implementation. Moreover, international conventions such as the Universal Declaration on Animal Welfare (62) advocate for the humane treatment of animals across all sectors, including livestock exhibitions and breeding (56). However, the absence of specific legal instruments targeting aesthetic tampering in camels creates a regulatory void that facilitates these unethical practices. Strengthening legal accountability through clear penalties, mandatory veterinary supervision, and cross-border cooperation is essential to address the dual concerns of animal welfare violations and fraudulent trade practices. Embedding legal awareness into community education programs and camel owner associations can also foster compliance and cultural shifts toward more humane practices.

## Effects of tampering on the society

Tampering in camels has far-reaching societal implications that go beyond its effects on animal health and commercial transactions, particularly in regions where camel husbandry is deeply intertwined with cultural identity, economic resilience, and food security. Such practices compromise the foundational values of trust, honesty, and integrity within the livestock trade, weakening relationships among buyers, sellers, and veterinary authorities (11). The resulting erosion of trust can lead to reduced participation in camel markets and a breakdown in social cohesion within pastoral and livestock-reliant communities. Additionally, the acceptance and perpetuation of unethical behavior promote a culture of dishonesty and diminish societal commitment to animal welfare, potentially influencing younger generations and reinforcing detrimental practices (10). Financial losses suffered by misled buyers may strain household economies and give rise to conflicts, further exacerbating social instability. Tampering also hinders effective veterinary monitoring and public health interventions, as altered physical traits can conceal signs of illness, increasing the risk of disease transmission to both animals and humans (9). To address these societal challenges, a comprehensive approach is required—one that incorporates educational initiatives, community involvement, strict ethical enforcement, and the development of transparent trading systems aimed at rebuilding trust, preserving cultural traditions, and supporting the long-term viability of camel-reliant communities (12, 16).

## Future directions toward tampering

To effectively address tampering in camels, future approaches should embrace a multidisciplinary and proactive strategy that combines scientific research, policy innovation, community involvement, and educational initiatives. Enhancing veterinary oversight through the adoption of standardized inspection protocols and mandatory certification for animals entering markets can play a crucial role in identifying and preventing tampering activities. Technological advancements, such as biometric identification and imaging methods, can further aid in the early detection and accurate documentation of physical alterations. Equally critical is the establishment of clear legal frameworks that define tampering offenses and impose penalties, along with robust enforcement practices. Public awareness campaigns at the community level are vital for shifting societal attitudes and fostering ethical behavior among camel owners, traders, and market participants. Integrating animal welfare education into professional training for veterinarians, pastoralists, and livestock traders will help cultivate a culture of responsibility and transparency. Additionally, international and regional cooperation is essential to align regulations and share best practices, especially in areas where camel trade is economically and culturally significant. By combining regulatory, educational, and technological measures, future efforts can effectively reduce tampering and promote the sustainability, welfare, and economic resilience of camel husbandry systems.

## Conclusion

In conclusion, the rising prevalence of illegal tampering in dromedary camels poses significant and complex challenges that affect animal welfare, economic stability, and societal well-being. Tampering, which involves deceptive physical alterations and modifications, has wide-ranging consequences for the health, productivity, and overall condition of camels, leading to both immediate and long-term negative effects. Beyond its impact on animal health, tampering disrupts market transparency, skews economic interactions, and erodes trust within camel trading networks. These practices also endanger public health by masking signs of disease and complicating veterinary supervision, while fostering unethical behavior that weakens social ties and cultural norms. To effectively address these issues, a comprehensive strategy is needed, one that includes stronger regulatory enforcement, the development of advanced detection technologies, and focused public awareness initiatives. Additionally, promoting collaboration among stakeholders at local, national, and international levels is key to minimizing the harmful effects of tampering and supporting sustainable camel husbandry. A collective commitment to ethical practices, underpinned by robust legal and educational frameworks, is vital to ensuring the long-term health and welfare of dromedary camels and the communities reliant on them.
